# Isolation, Synthesis and Structures of Ginsenoside Derivatives and Their Anti-Tumor Bioactivity

**DOI:** 10.3390/molecules15010399

**Published:** 2010-01-19

**Authors:** Mei Han, Jin-Gang Hou, Cheng-Ming Dong, Wei Li, Hao-Lun Yu, Yi-Nan Zheng, Li Chen

**Affiliations:** 1 College of Chinese Medicinal Materials, Jilin Agricultural University, Changchun 130118, China; E-Mail: houjingang1225@126.com (J.-G.H.); 2 Department of Pharmaceutical Science, Henan College of Traditional Chinese Medicine, Zhengzhou 450008, China; 3 Norman Bethune College of Medicine, Jilin University, Changchun, 130000, China

**Keywords:** isolation, synthesis, ginsenoside-ORh_1_

## Abstract

Protopanaxatriol saponins obtained with AB-8 macroporous resin mainly consisted of ginsenosides Rg_1_ and Re. A novel mono-ester of ginsenoside-Rh_1_ (ginsenoside-ORh_1_) was synthesized through further enzymatic hydrolysis and octanoyl chloride modifications. A 53% yield was obtained by a facile synthetic method. The structures were identified on the basis of 1D-NMR and 2D-NMR, as well as ESI-TOF-MS mass spectroscopic analyses. The isolated and synthetic compounds were applied in an anti-tumor bioassay, in which ginsenoside ORh_1_ showed moderate effects on Murine H22 Hepatoma Cells.

## Introduction

*Panax ginseng*, which belongs to the Araliaceae family, has been used as a traditional medicine for thousands of years and is now a popular natural medicine used worldwide [1,2]. Ginsenosides [3,4] have been regarded as the principal components responsible for the pharmacological activities of ginseng including anti-inflammatory activity, increasing the free radical scavenging activities and reducing weight [5,6,7,8,9]. Recent studies pointed that some rare ginsenosides such as Rh_1_, M1 had strong anticancer activity both *in vivo* and *in vitro* [10,11]. Pharmaceutical studies [12] have shown that ginseng saponins were ingested mainly by the bacteria of the small intestine and thus converted into their final forms: Re→Rg_1_→F_1_ or Rh_1_→M4; Rb_1_→Rd→F_2_→M1; Rb_2_→M6→M2→M1; Rc→M7→M3→M1. Previous studies also showed that ginsenoside-M1 was further esterified with fatty acids and thus could be sustained longer in the body, a result that suggested that the fatty acid ester of the M1 might be the real anti-tumor active species *in vivo* [13]. Our laboratory previously reported the synthesis of three novel mono-esters of ginsenoside-M1 (DM1, SM1, PM1) and their bioactivity [14]. According to the previous study, we established a method for synthesizing ORh_1_ and evaluated its bioactivity. Ginsenoside Rh_1_ was obtained through enzymatic hydrolysis and the fatty ester was modified by octanoyl chloride. The structure of ginsenoside-ORh_1_ was identified through ^1^H- and ^13^C-NMR and ESI-TOF-MS spectroscopic analyses. The pure ginsenoside-ORh_1_ was used to determine the anticancer bioactivity against Murine H22 Hepatoma Cells; the results showed that ginsenoside-ORh_1_ had moderate effects on H_22_ cells. This is the first time ginsenoside-ORh_1_ had been synthesized and reported. In this paper, we present the synthesis method and the bioactivity evaluation of the new ginsenoside fatty acid ester ORh_1_.

## Results and Discussion

### Characterizations of compounds ***1-4***

**Figure 1:**
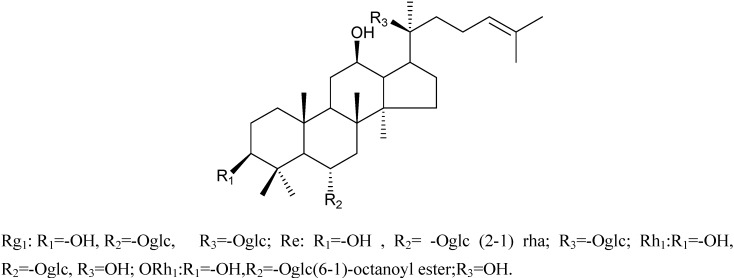
Isolated, enzyme produced and synthetic ginsenosides.

**Figure 2:**
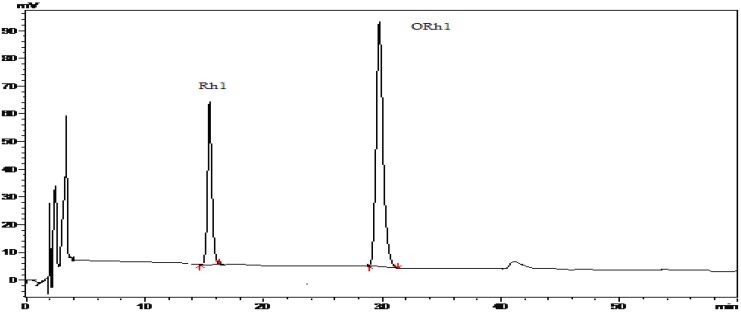
HPLC spectra of Rh_1_ (compound **3**) and the reaction product of ORh_1_ (compound **4**) HPLC conditions: isocratic elution with 100% MeOH for 60 min.

**Figure 3:**
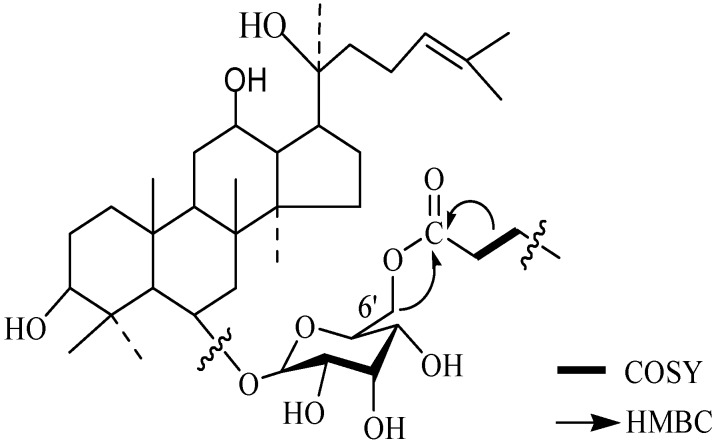
Partial HMBC and COSY correlation of the synthetic compound.

Compound **1** and compound **2** have been characterized as ginsenosides Rg_1_ and Re, whose structures were identified by MS and NMR data analysis (not shown). After an enzymatic hydrolysis process, we obtained ginsenoside Rh_1_ (Compound **3**). Then, ginsenoside Rh_1_ was modified by octanoyl chloride and formed ginsenoside fatty acid ester (compound 4). The structures of compounds **1-4** are shown in Figure 1. The HPLC analysis of compounds **3** and **4** is shown in Figure 2. From the HPLC analysis, the polarity of compound **4** was lower than that of Rh_1_ (compound **3**). From the ^13^C-NMR data analysis and comparison, we found no significant chemical shifts changes for the main skeleton but an upfield shift of C’-6 of the 6-O-Glu (δ 63.5 to δ 64.8, see Table 1), indicating that the fatty acid ester subsituent was connected to that position of 6-O-Glu was observed. This assumption had been verified by HMBC (see Figure 3) which showed a cross-peak between H-6’ to the carboxyl carbon.

**Table 1:** ^13^C-NMR Chemical Shifts(δ) of protopanaxatriol and compounds **3** and **4**. ^a^

No.(C)	Rh_1_	Compound 3	Compound 4
1	39.4	39.2	39.8
2	27.9	27.2	27.2
3	78.6	78.7	78.7
4	40.3	40.2	39.4
5	61.4	61.4	60.0
6	78.0	77.7	80.2
7	45.2	45.4	44.6
8	41.1	41.3	41.1
9	50.2	50.4	50.0
10	39.6	39.9	39.0
11	32.0	32.2	31.6
12	71.0	71.2	70.0
13	48.2	48.4	49.7
14	51.6	51.8	50.8
15	31.1	31.4	29.6
16	27.2	27.2	26.7
17	54.7	54.9	50.3
18	17.4	17.2	17.2
19	17.6	17.8	17.8
20	78.0	78.3	73.9
21	26.8	27.0	27.0
22	25.8	26.0	34.1
23	28.0	28.1	22.6
24	126.0	126.5	125.7
25	130.6	130.9	130.9
26	25.8	26.0	25.6
27	17.6	17.5	17.7
28	31.7	31.8	31.6
29	16.4	16.5	15.8
30	16.8	17.0	16.6

^a^ Compounds **3** and **4** were measured in CDCl_3_ and chemical shifts are expressed in ppm.

**Table 2:** ^13^C-NMR data of sugar and fatty acid ester moieties of compound **3** and **4**.

No.(C)	Rh_1_	Compound 3	Compound 4
1′	105.7	106.2	104.4
2′	75.4	75.6	74.0
3 ′	80.0	80.2	80.1
4′	71.8	72.0	72.0
5′	79.5	79.8	79.2
6′	63.5	63.3	64.8
1′′			174.3
2′′			34.1
3′′			24.9
4′′			30.3
5′′			29.1
6′′			30.9
7′′			22.6
8′′			14.1

^a^ Compounds 3 and 4 were measured in CDCl3 and chemical shifts are expressed in ppm.

### Bioactivity evaluation

**Figure 4:**
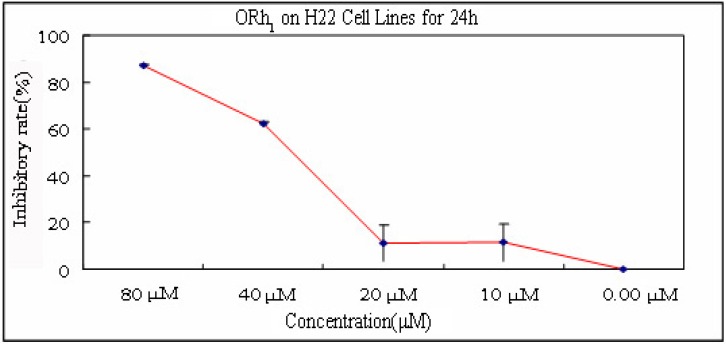
The rate of inhibition of ORh_1_ on H22 cell lines after 24 h.

The purified and synthetic compounds were tested in an anti-tumor bioassay. Compounds **3** and **4** showed moderate cytotoxicity effects against the Murine H22 Hepatoma Cells. The results are shown in Table 3 and Figure 4. We tested the sample for four concentrations (10 μM, 20 μM, 40 μM, 80 μM). The highest inhibitory rate of ORh_1_ was 87.16% at the concentration of 80 μM. The IC_50_ of ORh_1_ was obtained at the concentration of 42.44 μM.

**Table 3:** Anti-tumor activities of compound **3** and **4** (IC50 values in μM).

Concentration (μM)	Inhibitory rate (%)		IC_50_(μM)	
	Ginsenoside Rh_1_	ORh_1_	Ginsenoside Rh_1_	ORh_1_
0 μM	0	0	48.63	42.44
10 μM	8.97	11.44		
20 μM	9.28	11.09		
40 μM	54.21	62.34		
80 μM	76.54	87.16		

## Conclusions

The anti-tumor effects of some ginsenosides were known. For instance, Rg_3_ has been shown to possess anti-tumor properties and have an effect on drug-resistant cultured cancer cells [15,16]. Rh_2_ can reduce the proliferation of a variety of cultured cancer cells and can influence apoptosis [17,18,19]. Most of the ginsenosides with significant anti-tumor activities belongs to protopanaxdiol-type saponins. In our study, we investigated the protopanaxtriol-type ginsenoside Rh_1_. The protopanaxtriol-type saponins, including Rg_1_ and Re, were finally transformed into ginsenoside Rh_1_ after intestinal metabolism. We obtained the ginsenoside Rh_1_ by means of enzymolysis and further chemical modification led to the synthesis of Rh_1_ mono-fatty acid ester. The bioactivity evaluation of fatty acid ester (ORh_1_) showed that this kind of compound was more active against human tumor cells. We concluded that the membrane transportation of small molecules depends on their lipophilic abilities and the fatty acid ester (ORh_1_) with lower polarity met this requirement. In this study, we synthesized ORh_1_ with fatty acid acyl-chlorides and the synthetic product had the desired lower polarity. In this study, ORh_1_ showed more activity and higher anti-tumor efficiency than ginseonside Rh_1_ on Murine H22 Hepatoma Cells, and as time increased (more than 24 hr), the anti-tumor action of Rh_1_ was almost eliminated, whereas ORh_1_ still displayed considerable anti-tumor action. 

## Experimental

### General

The ^1^H- and ^13^C-NMR spectra were measured on a Bruker AV 400 NMR spectrometer in CDCl_3_, using TMS as an internal standard. Chemical shifts (*δ*) are expressed in parts per million (ppm). The HR-ESI-TOF mass spectra were obtained from a MDS SCIEX API QSTAR-MS instrument. Column chromatographies were carried out with silica gel 60M (200-300 mesh) and AB-8 macroporous resin and HPLC were carried out on an LC-2010 system (Shimadzu).

### Chemicals and reagents

Octanoyl chloride was purchased from ABCR GmbH& Co. KG. Enzyme (snailase) was purchased from BioDee BioTech Corporation Ltd. (code: S0100, Beijing, China). Other chemicals and reagents were purchased from the Chinese Chemical Group (Beijing, China).

### Extraction and isolation

Extracts of leaves from *Panax ginseng* (100 g) was dissolved with sufficient amount of distilled water and filtered, then the filtrate was absorbed on a AB-8 macroporous resin column (100 × 10 cm) for 8 h. Gradient elution with 25%, 30%, 80% ethanol was used to elute the column. The dry eluate was obtained from 30% ethanol fraction and weighted (45 g). The fraction consisted of the protopananxtriol-type saponins *Ginsenoside Rg_1_* (**1**), a white amorphous powder, mp 194–196 ^o^C; ESI-MS[+]: m/z=801 [M+H]^+^ and *GinsenosideRe* (**2**), a white amorphous powder, mp 201-203 ^o^C; ESI-MS[+]: m/z=969.3 [M+Na]^+^.

### Enzymatic reaction

A saponin fraction (1 g) obtained from the former procedure was weighed out and dissolved in distilled water (400 mL), enzyme (110 mg) was added and the mixture was cultured at 37 °C for 24 h while the pH was maintained at a value of 4.5. Then the enzyme reaction mixture was subjected to silica gel column chromatography eluting with CHCl_3_-MeOH (9:1) to afford purified *Ginsenoside Rh_1_* (**3**, 320 mg) as a white amorphous powder, mp 190–192 °C; ESI-MS[+]: m/z=639 [M+H]^+^; ^13^C-NMR data, see Table 1 and Table 2.

### Synthesis of Ginsenoside ORh1 *(**4**)*

**Scheme 1:**
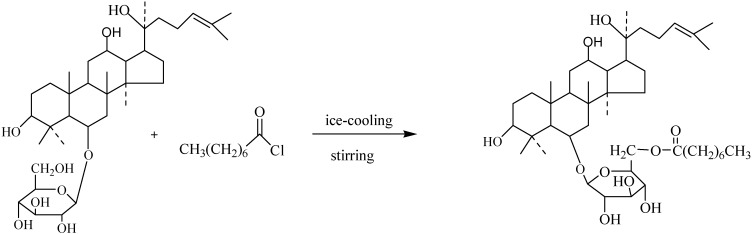
Preparation of ginsenoside ORh_1_(**4**).

Octanoyl chloride (125 mg) and K_2_CO_3_ (80 mg) were dissolved in CHCl_2_ (80 mL) under ice-cooling and stirred for 20min, then ginsenoside Rh_1_ (100 mg) dissolved in CHCl_2_ (1 mL) was added slowly. The mixture was reacted under stirring and with ice-cooling state for 24 h. The reacted solution was filtered through a 0.22 μm membrane, then the filtrate was condensed and subjected to silica gel column chromatography, eluted with CHCl_3_-MeOH=10:1 to give pure **4** (66 mg) as a colorless oil; HR-ESI-TOF-MS [+]^+^: m/z=764.2533 [M+H]^+^; ^13^C-NMR data, see Table 1 and Table 2. The purity was analyzed by HPLC (100%MeOH, Hypersil C_18_, 35 °C, 0.5 mL/min). The synthesis procedure is shown in Scheme 1.

### In vitro anti-tumor assays

Mono-nuclear cell direct cytotoxicity assay against human tumors cell lines were carried out at the Cell Culture Laboratory, Pharmaceutical College, Jilin University, using Murine H22 Hepatoma Cells. A blank control was used in this study. Generally, 5 × 10^5^/mL cells (190 μL) were placed in a 96-well plate and treated with obtained compound (10 μL). The normal cell was added culture medium. The plate was incubated at 37 °C for 24 h.Then, each well was added MTT (20 μL) with the concentration of 5 mg/mL and incubation at 37 °C was continued for 4 h. After that, the supernatant was removed and DMSO (150 μL) was added, the samples were agitated and the absorbance read at 490 nm.
